# Impact of anxiety and depression on the prognosis of copd exacerbations

**DOI:** 10.1186/s12890-022-01934-y

**Published:** 2022-04-29

**Authors:** Martínez-Gestoso Sandra, García-Sanz María-Teresa, Carreira José-Martín, Salgado Francisco-Javier, Calvo-Álvarez Uxío, Doval-Oubiña Liliana, Camba-Matos Sandra, Peleteiro-Pedraza Lorena, González-Pérez Miguel-Angel, Penela-Penela Pedro, Vilas-Iglesias Andrés, González-Barcala Francisco-Javier

**Affiliations:** 1 Emergencies Department, Salnés Couny Hospital, Vilagarcía de Arousa, Spain; 2 Translational Research in Airway Diseases Group (TRIAD)-Health Research Institute of Santiago de Compostela (IDIS), Santiago de Compostela, Spain; 3 Radiology Department, University of Santiago de Compostela, Santiago de Compostela, Spain; 4 Department of Biochemistry and Molecular Biology, Faculty of Biology-Biological Research Centre (CIBUS), Universidade de Santiago de Compostela, Santiago de Compostela, Spain; 5 Spanish Biomedical Research Networking Centre-CIBERES, Madrid, Spain; 6 Respiratory Medicine, Arquitecto Marcide Hospital, Ferrol, Spain; 7 University Hospital Complex of Santiago de Compostela, Santiago de Compostela, Spain; 8 Primary Care, CS Valle-Inclán, Ourense, Spain; 9 Respiratory Medicine, HM Hospital, Santiago de Compostela, Spain; 10 Clinic University Hospital Santiago de Compostela, Santiago de Compostela, Spain; 11 Department of Medicine, University of Santiago de Compostela, Santiago de Compostela, Spain

**Keywords:** COPD, Anxiety, Depression, Prognosis

## Abstract

**Background:**

Frequent and highly prevalent as comorbidities in Chronic Obstructive Pulmonary Disease (COPD) patients, both depression and anxiety seem to have an impact on COPD prognosis. However, they are underdiagnosed and rarely treated properly.

**Aim:**

To establish the prevalence of depression and anxiety in patients admitted for Acute Exacerbation of COPD (AECOPD) and determine their influence on COPD prognosis.

**Methods:**

Prospective observational study conducted from October 1, 2016 to October 1, 2018 at the following centers in Galicia, Spain: Salnés County Hospital, Arquitecto Marcide, and Clinic Hospital Complex of Santiago de Compostela. Patients admitted for AECOPD who agreed to participate and completed the anxiety and depression scale (HADS) were included in the study.

**Results:**

288 patients (46.8%) were included, mean age was 73.7 years (SD 10.9), 84.7% were male. 67.7% patients were diagnosed with probable depression, and depression was established in 41.7%; anxiety was probable in 68.2% and established in 35.4%. 60.4% of all patients showed symptoms of both anxiety and depression. Multivariate analysis relates established depression with a higher risk of late readmission (OR 2.06, 95% CI 1.28; 3.31) and a lower risk of mortality at 18 months (OR 0.57, 95% CI 0.37; 0.90).

**Conclusion:**

The prevalence of anxiety and depression in COPD patients is high. Depression seems to be an independent factor for AECOPD, so early detection and a multidisciplinary approach could improve the prognosis of both entities. The study was approved by the Ethical Committee of Galicia (code 2016/460).

## Introduction

The multidisciplinary approach to patients is particularly relevant in highly prevalent chronic diseases, such as chronic obstructive pulmonary disease (COPD). With COPD, as with other chronic diseases, it is common for comorbidities to worsen patients’ quality of life, to interfere with the perception of other symptoms and to worsen prognosis [1–5]. Depression and anxiety are frequent comorbidities in COPD patients, with an estimated prevalence of 8–80% and 2–96%, respectively [6, 7].

Various protocols advise evaluating anxiety and depression in COPD patients, especially the most severe or exacerbating ones [8–10]. COPD patients show symptoms of depression and anxiety more frequently than the general population, and it seems that both entities have an impact on prognosis, as physical activity is reduced, dyspnea is worsened, the frequency of exacerbations increase and so does the use of health resources. Moreover, depression and anxiety interfere with other risk factors, such as tobacco use, and, in general, they impair patients’ quality of life [3, 11]. However, the debate on the impact of anxiety and depression on the prognosis of COPD patients continues, as some authors report worse disease progression [12–14], while other researchers have not observed any association between anxiety or depression and worse prognosis of acute exacerbations of COPD (AECOPD) [15, 16]. Also, though some studies have analyzed the relationship between depression and anxiety with COPD, both entities are underdiagnosed in these patients and therefore rarely treated properly [17].

Our objective is to establish the prevalence of depression and anxiety in patients admitted for AECOPD and determine their influence on prognosis.

## Methods

Prospective observational study conducted from October 1, 2016 to October 1, 2018 at the following centers in Galicia, Spain: Salnés County Hospital (Vilagarcía de Arousa), Arquitecto Marcide (Ferrol) and Clinic Hospital Complex of Santiago de Compostela. Patients admitted for AECOPD who agreed to participate and signed the informed consent form were included in the study. Patients with AECOPD were identified through the admission records of the participating hospitals, reviewed daily during the study period. The participation of all consecutive patients was requested, except in cases with a history of advanced cognitive impairment, which were excluded. The medical records of the patients were reassessed one and a half years after the end of the investigation period to determine the number of readmissions and mortality. Diagnosis, baseline severity, and AECOPD were defined following the GOLD criteria [8]. Demographic and descriptive variables were obtained from the computerized clinical history of each patient and through an interview during admission. Approximately 20% of patients lacked spirometry; where it was available, the most recent performed at baseline was used. Early readmission was defined as that occurring within the first 15 days following discharge from the index admission, and late readmission was defined as that occurring from the 16th day following discharge to the completion of the study [18]. Symptoms of anxiety and depression were identified with the hospital anxiety and depression scale (HADS) [19]. The HADS scale was administered by interview, by the members of the research team, to those patients admitted for COPD who agreed to participate. Possible depression was considered for those patients scoring ≥ 8, and probable depression for those scoring ≥ 11 on the HADS Depression subscale. Similarly, possible anxiety was considered for patients scoring ≥ 8 on the HADS Anxiety subscale, and probable anxiety for those scoring ≥ 11 on the HADS Anxiety subscale [20].

### Statistical analysis

The data obtained are expressed as mean ± standard deviation (SD) in continuous variables, and as frequencies and percentages in categorical variables. Continuous variables were compared using Student's t test or Wilcoxon test; in the case of categorical variables, the chi-square test and Fisher’s exact test were used. Multivariate and univariate analyses were performed. The relationship of anxiety and depression with readmission and mortality was determined by Cox regression, adjusted for age, sex and lung function, in addition to severe exacerbations in the previous year, counted as admissions or visits to the hospital emergency department due to COPD. The analyses were carried out with SPSS 15.

## Results

During the study period, 615 patients admitted for AECOPD agreed to participate, and 288 patients (46.8%) completed the HADS questionnaire and were included in the study. Mean age was 73.7 years (SD 10.9) and 84.7% were male. 67.7% were identified with possible depression, and depression was probable in 41.7%; anxiety was possible in 68.2% and probable in 35.4%. 60.4% showed symptoms of both anxiety and depression. Mean stay was 6.8 days (SD 5.6). Hospital mortality was 1.4%. During the study period, 18 patients (6.3%) were readmitted early, and 41% were readmitted late. Mortality at 18 months was 47%. Baseline characteristics of the study population are shown in Table 1.

**Table 1:** Characteristics of the study population

Mean age, years (SD)	73.7 (10.9)
Sex, n (%)	
Male	244 (84.7)
Female	44 (15.3)
Tobacco use, n (%)	
Active smoker	81 (28.1)
Former smoker	177 (61.5)
Never smoker	19 (6.6)
Unknown	11 (3.8)
FEV1%, mean (SD)	53.0 (19.6)
FEV1/FVC, mean (SD)	50.5 (13.6)
GOLD, n (%)	
Mild	22 (9.6)
Moderate	97 (42.4)
Severe	89 (38.9)
Very severe	21 (9.2)
BMI, kg/m^2^, mean (SD)	28.9 (5.8)
ICU, n (%)	1 (0.3)
Admissions previous year, n (%)	
0	173 (60.1)
1	52 (18.1)
≥ 2	63 (21.9)
ED previous year, n (%)	
0	117 (40.6)
1	60 (20.8)
≥ 2	111 (38.5)
Early readmission, n (%)	18 (6.3)
Late readmission, n (%)	118 (41)
Hospital mortality, n (%)	4 (1.4)
Mortality at 18 months, n (%)	135 (47)
HADS-anxiety, n (%)	
≥ 8 points	197 (68.4)
≥ 11 points	102 (35.4)
HADS-depression, n (%)	
≥ 8 points	195 (67.7)
≥ 11 points	120 (41.7)

BMI, body mass index; ED, emergency department; FEV1, forced expiratory volume in the first second; FVC, forced vital capacity; HADS, hospital anxiety and depression scale; SD, standard deviation; UCI, intensive care unit

Univariate analysis shows a higher probability of late readmission and lower mortality at 18 months in patients with anxiety and depression (Table 2). Multivariate analysis relates probable depression with a higher risk of late readmission (OR 2.06, 95% CI 1.28; 3.31) and a lower risk of mortality at 18 months (OR 0.57, 95% CI 0.37; 0.90). No significant relationship between probable anxiety and prognosis was found (Table 3). No significant relationship between possible depression/anxiety and prognosis was found (data not shown).

**Table 2:** Probability of readmission and mortality in relation to anxiety or depression. Univariate analysis

	Anxiety 0–7	Anxiety 8–10	Anxiety ≥ 11	p
Early readmission, n (%)				0.36
Yes	3 (3.3)	7 (7.4)	8 (7.8)	
No	88 (96.7)	88 (92.6)	94 (92.2)	
Late readmission, n (%)				0.01
Yes	27 (29.7)	49 (51.6)	42 (41.2)	
No	64 (70.3)	46 (48.4)	60 (58.8)	
Mortality at 6 months, n (%)				0.58
Yes	12 (13.2)	15 (15.8)	19 (18.6)	
No	79 (86.8)	80 (84.2)	83(81.4)	
Mortality at 12 months, n (%)				0.32
Yes	17 (18.7)	26 (27.4)	21 (20.8)	
No	74 (81.3)	69 (72.6)	80 (79.2)	
Mortality at 18 months, n (%)				0.03
Yes	53 (58.2)	41 (43.2)	41 (40.6)	
No	38 (41.8)	54 (56.8)	60 (59.4)	

	Depression 0–7	Depression 8–10	Depression ≥ 11	p
Early readmission, n (%)				0.3
Yes	3 (3.2)	5 (6.7)	10 (8.3)	
No	90 (96.8)	70(93.3)	110 (91.7)	
Late readmission, n (%)				0.008
Yes	26 (28)	36 (48)	56 (46.7)	
No	67 (72)	39 (52)	64 (53.3)	
Mortality at 6 months, n (%)				0.45
Yes	12 (12.9)	15 (20)	19 (15.8)	
No	81 (87.1)	60 (80)	101 (84.2)	
Mortality at 12 months, n (%)				0.2
Yes	15(16.1)	20 (26.7)	29 (24.4)	
No	78 (83.9)	55 (73.3)	90 (75.6)	
Mortality at 18 months, n (%)				0.001
Yes	52 (63.4)	29 (38.7)	47 (39.5)	
No	34 (36.6)	46 (61.3)	72 (60.5)	

Fisher's test was applied to the variable “early readmission” as an alternative to chi-square, because one of the cell counts in the table was less than 5

**Table 3:** Probability of readmission and mortality in relation to anxiety or depression. Multivariate analysis

	Early readmission	Late readmission	Mortality at 18 months
Anxiety OR (95% CI)			
HADS-A < 11	1	1	1
HADS-A ≥ 11	1.01 (0.26;3.91)	1.27 (0.81;2.00)	0.70 (0.98;1.00)
Depression OR (95% CI)			
HADS-D < 11	1	1	1
HADS-D ≥ 11	0.67 (0.16;2.75)	2.06 (1.28;3.31)	0.57 (0.37;0.90)

Adjusted by age, gender, FEV1, admissions previous year, emergency department previous year

## Discussion

The data reported by different studies offer a wide range of estimations of the prevalence of depression and anxiety in COPD patients, probably due to differences not only across the different populations, but also in the scales and tools used in the diagnosis. Various risk factors have been identified for the development of anxiety and depression in COPD patients, including severe dyspnea, a history of tobacco use, the presence of other comorbidities, a low educational level, a low socioeconomic status and, overall, a lower quality of life [21]. The prevalence of anxiety and depression is high among our patients, and higher than that of COPD patients in some previous studies [7, 22, 23, 25, 26]. These differences could be explained in part by the older age of our population or by the lower percentage of males included by other authors [7, 23]. However, our figures are similar to those reported by Phan et al. when both entities are presented jointly [27]. Also, the presence of depressive symptoms in COPD patients is associated with an increase in severe exacerbations, a reduction in physical activity, an increase in dyspnea, and a deterioration in quality of life [3, 11, 26], which suggests that depression worsens the progression of COPD.

A recent study has shown that the prevalence of depression in COPD is higher in frequent exacerbators and that depression is more severe in patients at a higher COPD stage [12]. In our population, patients with depression have a higher risk of readmission for AECOPD, regardless of lung function and severe exacerbations in the previous year, evaluated as attending the Emergency Department or being admitted to hospital. In a systematic review, Lecheler et al. reported high readmission rates in patients with depression hospitalized for AECOPD [13]. In a retrospective study conducted with hospitalized patients, Iyer et al. found an association between depression and readmission evaluated at 30, 90 and 365 days [28]. Similar to our results, other authors have associated depression with readmission, but without a significant relationship with anxiety [7, 29]. The meta-analysis by Laurin et al. has also shown an association between depression and risk of AECOPD, but not anxiety [30]. Other authors, however, have reported a higher risk of AECOPD in patients with anxiety [31]. The differences in results across studies could be related to the heterogeneity of the populations studied and the variability in the definition of COPD (changes in symptoms and treatment, inclusion of outpatients versus inpatients, comorbidities considered…), as well as the various methods used for the evaluation of anxiety and depression.

COPD exacerbations seem to indicate a high risk of mortality, exceeding 26% in the year following an exacerbation that requires hospital admission [4]. Various authors have related anxiety and depression with an increased risk of mortality after hospital discharge [13, 14]. In our study, we have found no relationship between anxiety and depression with mortality, but surprisingly, mortality at 18 months is lower in patients with depression. Previous studies found a slight protective effect of anxiety on hospital mortality [15] or higher anxiety in women than in men in COPD patients, but no differences in mortality at 3 years after adjusting by age and FEV1 [32].

In the study by Zilz et al., depression and anxiety are not associated with survival in severe COPD [33]. There is also no relationship between severe depression and mortality in stable COPD in the study by Maters et al. [16]. The care received after admission may have been different and the controls may have been more exhaustive in patients with COPD and established depression, leading to a lower mortality rate in this group. Unfortunately, this information is not available, so we do not have a solid explanation for the unexpected finding.

The pathophysiological mechanisms that explain the effect of depression on AECOPD are not well known, although there appear to be common symptoms and a bidirectional relationship between the two: COPD increases the risk of depression, and patients with COPD show symptoms of depression and anxiety more frequently than the general population [3]. An interaction among psychophysiological, behavioral, and psychosocial causes has been suggested. Depression entails feelings of helplessness, isolation, hopelessness, and fear that lead to loss of self-confidence, disinterest in self-care, poorer adherence to treatment, and a higher probability of continuing to smoke [11, 34], contributing to AECOPD. The impairment of cognitive functions appearing along depression may lead to higher perception of dyspnea, increasing the use of health services, and thus the possibility of admission. Depression is associated with chronic stress, which leads to sustained activation of the sympathetic nervous system and an increase in the systemic inflammatory response; both may compromise the immune system, favoring infections and increasing the frequency of exacerbations [30, 35]. The perception of depressed mood as a normal reaction to suffering from a chronic and incurable disease may perpetuate the symptoms, by failing to actively search for mood alterations as part of the diagnostic and therapeutic plan for these patients [34]. Episodes of AECOPD, which often lead to hospitalization, contribute in turn to hopelessness and depressed mood, thus closing the circle [36].

Our study has some limitations: first, only 46.8% of those admitted for AECOPD completed the evaluation questionnaire, which could result in bias in the estimation of anxiety and depression in this group. The profile of those who refused to complete the scale was more severe AECOPD (admitted to the ICU, exacerbation of bacterial cause or with pneumonia, those subjected to mechanical ventilation and non-invasive mechanical ventilation), more AECOPD in the previous year and more advanced stages of COPD disease (GOLD 3 and 4). Second, the HADS scale was used; despite being frequently used and validated for COPD patients, it does not seem to clearly discriminate between depression and anxiety [37], which could partially explain the differences in the results from other studies. Third, we ignore any potential variations in the self-perception of anxiety or depression during the study period, since the evaluation of symptoms was done upon index admission only. We also lack information about any treatment or interventions aimed at improving anxiety and depression in these patients, so we cannot explain what other variables have influenced the prognosis. Fourth, the predominance of men in the study corresponds to the prevalence of COPD by sex in Spain [38]. This could affect the interpretation of the results. However, the multivariate analyzes were adjusted for sex, which eliminates the possible gender bias. Finally, AECOPD was considered for patients admitted to hospital only, so the number of exacerbations may be underestimated: those treated by the Emergency Department or in Primary Care have not been evaluated.

In conclusion, the prevalence of anxiety and depression in COPD patients is high. Depression seems to be an independent factor for AECOPD, so early detection and a multidisciplinary approach could improve the prognosis of both entities.

## Data Availability

The datasets generated and/or analysed during the current study are not publicly available because this database is being used in another work as part of a doctoral thesis, so we would prefer that, before its completion, it was not for public use. Dr. García-Sanz is the guarantor of the paper and all information about this (database and informed consents signed by patients). Access to this information could be provided in the future, duly justifying it, since there is another study pending completion based on these data.
