# Correction to: Impact of anxiety and depression on the prognosis of copd exacerbations

**DOI:** 10.1186/s12890-022-01999-9

**Published:** 2022-05-30

**Authors:** Sandra Martínez-Gestoso, María-Teresa García-Sanz, José-Martín Carreira, Francisco-Javier Salgado, Uxío Calvo-Álvarez, Liliana Doval-Oubiña, Sandra Camba-Matos, Lorena Peleteiro-Pedraza, Miguel-Angel González-Pérez, Pedro Penela-Penela, Andrés Vilas-Iglesias, Francisco-Javier González-Barcala

**Affiliations:** 1 Emergencies Department, Salnés Couny Hospital, Vilagarcía de Arousa, Spain; 2 Translational Research in Airway Diseases Group (TRIAD)-Health Research Institute of Santiago de Compostela (IDIS), Santiago de Compostela, Spain; 3 Radiology Department, University of Santiago de Compostela, Santiago de Compostela, Spain; 4 Department of Biochemistry and Molecular Biology, Faculty of Biology-Biological Research Centre (CIBUS), Universidade de Santiago de Compostela, Santiago de Compostela, Spain; 5 Spanish Biomedical Research Networking Centre-CIBERES, Madrid, Spain; 6 Respiratory Medicine, Arquitecto Marcide Hospital, Ferrol, Spain; 7 University Hospital Complex of Santiago de Compostela, Santiago de Compostela, Spain; 8 Primary Care, CS Valle-Inclán, Ourense, Spain; 9 Respiratory Medicine, HM Hospital, Santiago de Compostela, Spain; 10 Clinic University Hospital Santiago de Compostela, Santiago de Compostela, Spain; 11 Department of Medicine, University of Santiago de Compostela, Santiago de Compostela, Spain

## Correction to: BMC Pulmonary Medicine (2022) 22:169 https://doi.org/10.1186/s12890-022-01934-y

Following publication of the original article [1], it was flagged that the authors' respective given and family names had been erroneously swapped in the author list. The author list has since been corrected in the published article and the correct list may be seen in this erratum.
